# Words for the Wise

**Published:** 1887-06-11

**Authors:** 


### WORDS FOR THE WISE.

The healing art can boast no home
More fraught with zeal and knowledge
Than that whose shadow sweeps the dome
Of University College.
She, midst a goodly sister band,
Though young, yet for rebuilding
Bespeaks a widely opened hand—
Not for mere paint and gilding.

Aright to spread her garnered love,
To cope with deadly scourges,
More liberal housing needs, much more—
So keen experience urges—
Of kindly-tempered, purest air,
With cheerful light, unswerving—
Unstinted be your help ; nor spare
Till aid o'ertakes deserving !—OSTRICH.

